# Milk consumption and health outcomes in Western and Asian populations

**DOI:** 10.1017/S1368980026103127

**Published:** 2026-07-14

**Authors:** Zui C. Narita, Japanese National Center Cohort Collaborative for Advancing Population Health Group

**Affiliations:** 1 National Center of Neurology and Psychiatry

**Keywords:** Dietary behaviour, Nutrition, Epidemiology, Public health

## Abstract

The Dietary Guidelines for Americans recommend three cup equivalents of dairy per day, whereas the Nutrition Source recommends limiting consumption to one to two servings per day. The evidence underlying these recommendations is largely derived from studies conducted in Western populations with high baseline milk consumption. This commentary discusses evidence on milk consumption and health outcomes in Western and Asian populations. Differences across studies appear to be influenced not only by racial or ethnic background but also by baseline milk consumption, residual confounding and differences in the populations studied. Dietary guidance should consider population-specific baseline intake and contextual factors.

Milk is widely consumed, particularly in Western countries. The Dietary Guidelines for Americans recommend three cup equivalents of dairy per day^(1)^. In contrast, the Nutrition Source, an educational nutrition website from the Harvard School of Public Health, recommends limiting consumption to one to two servings per day, citing potential risks associated with milk consumption^(2)^. The recommendation by the Nutrition Source is based on previous studies, but these studies were primarily conducted in the USA and predominantly involved White participants, with only limited representation of Asian populations across low- to high-income settings. Milk consumption varies substantially by country, culture and ethnicity, with Asians typically consuming less^(3,4)^. In many cases, evidence of milk consumption comes from cohort studies that compare groups with higher milk consumption to those with lower milk consumption. Hence, the OR, risk ratio or hazard ratio derived from these studies is substantially influenced by the odds, risk and hazard of the lower consumption group. For Asian populations, it is conceivable that the impact of milk consumption differs from that in other populations, not only due to racial and genetic factors but also because of differences in average consumption patterns. This article compares past research on milk consumption and health outcomes, primarily from Western populations, with evidence from Asian populations to discuss effect heterogeneity of milk consumption. Because ‘dairy’ encompasses diverse products with varying health effects (e.g. butter, yogurt), analysing them together can be misleading. Therefore, this commentary specifically isolates milk to provide a clearer understanding of its health implications.

## Effect heterogeneity across clinical outcomes

### CVD

The Nutrition Source cites past research on milk consumption and CVD. One US study analysed combined data from the Nurses’ Health Study, Nurses’ Health Study II and Health Professionals Follow-up Study, reporting higher CVD mortality with whole milk intake^(5)^. In this study, the lowest consumption groups (Q1) had mean intakes of 0·52, 0·67 and 0·96 servings per day across the three samples (one serving = 240 ml), while the highest consumption groups (Q5) had mean intakes of 4·00, 4·09 and 6·43 servings per day, respectively. On the other hand, a Japanese study reported an association between milk consumption and a lower hazard of CVD-related mortality in males, whereas evidence was not strong in females^(4)^. In this study, the lowest consumption group had a mean intake of only 0·5 grams per day in males and 2·6 grams per day in females, while the highest consumption group had mean intakes of 272·8 grams per day in males and 305·4 grams per day in females. Thus, both the lowest and highest milk consumption levels differed markedly across these studies. A meta-analysis that included both Asian and White populations found that consuming 200 ml of milk per day was most protective against stroke^(6)^. A recent study using the China Kadoorie Biobank found that regular dairy consumption (four or more times per week) was associated with a higher hazard of CHD but a lower hazard of stroke compared with never or rare consumption^(3)^. The heterogeneous findings on milk consumption appear to be explained not only by racial or ethnic differences but also by the actual levels of intake in low- and high-consumption groups. Thus, certain populations with lower milk consumption may benefit from increased milk intake. Other factors, such as residual confounding, may have also influenced the results. Moreover, the heterogeneous findings may reflect differences in the populations studied. For instance, the Nurses’ Health Study, Nurses’ Health Study II and Health Professionals Follow-up Study cohorts all consisted of medical professionals. Furthermore, there may be inconsistencies between the development of CHD and stroke, which could have affected the findings.

### Type 2 diabetes mellitus

The Nutrition Source cites a study showing that both skimmed milk and whole milk were associated with a higher risk of type 2 diabetes mellitus (T2DM)^(7)^. However, these findings were again based on the Nurses’ Health Study, Nurses’ Health Study II and Health Professionals Follow-up Study, which involved populations with higher baseline milk consumption. A meta-analysis of observational studies found that total milk consumption was associated with a higher risk of T2DM in European populations but a lower risk in the Asian population^(8)^. This meta-analysis included a Japanese study, in which women who consumed more than 200 ml of milk per day had an OR of 0·87 for T2DM compared with women consuming less than 50 ml, although the association did not reach statistical significance^(9)^. Another study enrolling Chinese individuals found that daily milk drinkers had a lower hazard of T2DM compared to non-drinkers^(10)^. The median milk consumption in this study was only 20·5 grams per day, and 67 % of participants never or rarely drank milk. As with CVD, baseline consumption appears to influence the impact of milk consumption, and certain populations, particularly those with low milk consumption, may benefit from drinking milk. Confounding and differences in the populations studied, similar to those discussed in the CVD section, are conceivable.

While milk consumption might be associated with various types of cancer, we focus on prostate and colorectal cancers, as these are specifically discussed in the Nutrition Source^(2)^.

### Prostate cancer

The evidence regarding milk consumption and prostate cancer has been controversial. The Nutrition Source cites a study analysing the Physicians’ Health Study, which showed an association between whole milk intake and a higher hazard of fatal prostate cancer^(11)^. On the other hand, a recent meta-analysis showed that whole milk consumption was associated with a lower risk of prostate cancer, whereas total milk consumption was linked to a higher risk^(12)^. Such inconsistencies might be due to differences in the populations studied; for example, the Physicians’ Health Study included only physicians, who are likely to have healthier lifestyles and lower baseline risks. Also, confounding bias, such as the lack of adjustment for physical activity, might have influenced the results. Regarding Asian populations, past research on Japanese men found an association between milk consumption and a higher likelihood of prostate cancer^(13)^. Given these inconsistent findings, it is not possible to confidently endorse or discourage milk consumption in relation to prostate cancer risk.

### Colorectal cancer

The Nutrition Source cites a meta-analysis indicating that milk consumption is associated with a lower risk of colorectal cancer^(14)^. A recent meta-analysis reported similar findings, suggesting an association between milk consumption and a lower risk of colorectal cancer^(15)^. Relative risks were reduced across studies from the USA, Europe and Asia, although only a single study from Asia was included. A more recent case–control study conducted in China found that milk drinkers had lower odds of developing colorectal cancer than non-drinkers^(16)^. Taken together, milk consumption may have an overall favourable association with colorectal cancer risk, particularly in Western populations, with a similar association also plausible in Asian populations.

### Cognition

While not explicitly evaluated in the Nutrition Source, cognition is a frequently analysed outcome in relation to milk consumption. A recent meta-analysis examining the association between milk consumption and cognition, which included five studies, found no significant difference between the highest and lowest consumption groups^(17)^. In that meta-analysis, the studies from Western settings were limited. On the other hand, a dose–response analysis solely based on three Asian studies showed that higher milk intake was associated with a lower risk of cognitive decline or dementia. The findings remain inconclusive and warrant further research.

## Discussion and conclusions

Although milk intake has been reported to be associated with adverse outcomes in populations with relatively high baseline consumption, small or moderate increases in intake may favourably affect health outcomes in populations with low baseline milk consumption (e.g. around 50 ml/d or less). The inconsistencies observed in previous studies could be largely explained by differences in baseline milk consumption, differences in the populations studied and residual confounding. The evidence appears most consistent for colorectal cancer, opposite in direction for CVD and T2DM across Western and Asian populations, inconsistent for prostate cancer and limited and inconclusive for cognition. Further research is needed particularly for prostate cancer and cognition. Evidence from Asian populations beyond Japan and China (such as India) is also warranted. This commentary highlights the importance of considering demographic-specific evidence when assessing the health implications of milk consumption. Current association studies cannot yet clarify genetic differences, optimal milk intake or environmental implications. Future research, including mediation analyses^(18)^ to decompose genetic and milk intake-related effects, interaction analyses^(19)^ between milk intake and environmental factors, and machine learning approaches^(20)^ to better assess heterogeneity in optimal milk intake, may help address these limitations.

In conclusion, milk consumption should be interpreted in relation to baseline intake and population context rather than through a uniform recommendation across populations. An individualised and balanced approach to milk consumption remains the most prudent dietary strategy.
